# Conceptual comparison of constructs as first step in data harmonization: Parental sensitivity, child temperament, and social support as illustrations

**DOI:** 10.1016/j.mex.2022.101889

**Published:** 2022-10-26

**Authors:** Marije L. Verhage, Carlo Schuengel, Annaleena Holopainen, Marian J. Bakermans-Kranenburg, Annie Bernier, Geoffrey L. Brown, Sheri Madigan, Glenn I. Roisman, Mette S. Vaever, Maria S. Wong, The Collaboration on Attachment Transmission Synthesis (CATS), Marian J. Bakermans-Kranenburg, Lavinia Barone, Kazuko Y. Behrens, Johanna Behringer, Annie Bernier, Ina Bovenschen, Geoffrey L. Brown, Rosalinda Cassibba, Jude Cassidy, Gabrielle Coppola, Alessandro Costantini, Mary Dozier, Karin Ensink, R. M. Pasco Fearon, Brent Finger, Airi Hautamaki, Nancy L. Hazen, Elena Ierardi, Inês Jongenelen, Simo Køppe, Francesca Lionetti, Sheri Madigan, Sarah Mangelsdorf, Mirjam Oosterman, Cecilia S. Pace, K. Lee Raby, Cristina Riva Crugnola, Glenn I. Roisman, Carlo Schuengel, Alessandra Simonelli, Gottfried Spangler, George M. Tarabulsy, Mette S. Væver, Marije L. Verhage, Maria S. Wong, Bronia Arnott, Heidi Bailey, Patrick J. Brice, Karl-Heinz Brisch, Germana Castoro, Elisabetta Costantino, Chantal Cyr, Carol George, Gabriele Gloger-Tippelt, Sonia Gojman, Susanne Harder, Carollee Howes, Heidi Jacobsen, Deborah Jacobvitz, Mi Kyoung Jin, Femmie Juffer, Miyuki Kazui, Esther M. Leerkes, Karlen Lyons-Ruth, Catherine McMahon, Elizabeth Meins, Salvador Millán, Lynne Murray, Katja Nowacki, David R. Pederson, Lynn Priddis, Avi Sagi-Schwartz, Sarah J. Schoppe-Sullivan, Judith Solomon, Anna Maria Speranza, Miriam Steele, Howard Steele, Doug M. Teti, Marinus H. van IJzendoorn, W. Monique van Londen-Barentsen, Mary J. Ward

**Affiliations:** a Vrije Universiteit Amsterdam, The Netherlands; b University of Pavia, Italy; c SUNY Polytechnic Institute, Utica, NY; d Friedrich-Alexander-Universität Erlangen-Nürnberg, Germany; e University of Montréal, Canada; f University of Erlangen - Nuremberg, Germany; g University of Georgia, GA; h University of Bari Aldo Moro, Italy; i University of Maryland, College Park, MD; j University of Bari Aldo Moro, Italy; k University of Bari Aldo Moro, Italy; l University of Delaware, Newark, DE; m Université Laval, Quebec City, Canada; n University College London, UK; o Montana State University Billings, MT; p University of Helsinki, Finland; q University of Texas at Austin, TX; r University of Milano-Bicocca, Italy; s Universidade Lusófona do Porto, Portugal; t University of Copenhagen, Denmark; u d'Annunzio University of Chieti-Pescara, Italy and Queen Mary University of London, UK; v University of Calgary and the Alberta Children's Hospital Research Institute, Calgary, Canada; w University of Wisconsin-Madison, WI; x Vrije Universiteit Amsterdam, The Netherlands; y University of Genoa, Italy; z University of Utah, Salt Lake City, UT; aa University of Milano-Bicocca, Italy; bb University of Minnesota, Minneapolis, MN; cc Vrije Universiteit Amsterdam, The Netherlands; dd University of Padova, Italy; ee University of Erlangen - Nuremberg, Germany; ff Université Laval, Quebec City, Canada; gg University of Copenhagen, Denmark; hh Vrije Universiteit Amsterdam, The Netherlands; ii Endicott College, MA.; jj Newcastle University, UK; kk University of Guelph, Canada; ll Gallaudet University, Washington, DC; mm Paracelsus Medical School, Salzburg, Austria; nn University of Bari "Aldo Moro", Italy; oo University of Milano-Bicocca, Italy; pp Université du Québec à Montréal and CIUSSS Centre-Sud-de-l’île de Montréal, Canada; qq Mills College, Oakland, CA; rr Ulm University Medical Center, Germany; ss Research Center of the Seminario de Sociopsicoanálisis, Mexico City, Mexico; tt University of Copenhagen, Denmark; uu University of California at Los Angeles, CA; vv Region Centre for Child and Adolescent Mental Health, Eastern and Southern Norway, Norway; ww University of Texas at Austin, TX; xx Soomkyung Women's University, Seoul, South Korea; yy Leiden University, The Netherlands; zz Ibaraki University, Mito, Japan; aaa University of North Carolina at Greensboro, NC; bbb Harvard Medical School, Cambridge, MA; ccc Macquarie University, Sydney, Australia; ddd University of York, UK; eee Research Center of the Seminario de Siciopsicoanálisis, Mexico City, Mexico; fff University of Reading, UK; ggg Fachhochschule Dortmund, Germany; hhh Western University, London, Ontario, Canada; iii Edith Cowan University, Western Australia; jjj University of Haifa, Israel; kkk Ohio State University, Columbus, OH; lll Cambridge University School of Medicine, UK; mmm Sapienza University of Rome, Italy; nnn The New School for Social Research, New York, NY; ooo The New School for Social Research, New York, NY; ppp The Pennsylvania State University, State College, PA; qqq Erasmus University Rotterdam, The Netherlands; rrr Utrecht University, The Netherlands; sss Weill Cornell Medical College, New York, NY; a Clinical Child and Family Studies, Vrije Universiteit Amsterdam, The Netherlands; b Amsterdam Public Health Research Institute, Vrije Universiteit Amsterdam, The Netherlands; c Department of Psychology, University of Montréal, Canada; d Human Development and Family Science, University of Georgia, USA; e Department of Psychology, University of Calgary, Canada; f Alberta Children's Hospital Research Institute, Calgary, Alberta, Canada; g Institute of Child Development, University of Minnesota, USA; h Department of Psychology, University of Copenhagen, Denmark; i Endicott College, Beverly, MA, USA

**Keywords:** Data harmonization, Individual participant data meta-analysis, Construct validity, Data pooling, IPD, Individual participant data

## Abstract

This article presents a strategy for the initial step of data harmonization in Individual Participant Data syntheses, i.e., making decisions as to which measures operationalize the constructs of interest - and which do not. This step is vital in the process of data harmonization, because a study can only be as good as its measures. If the construct validity of the measures is in question, study results are questionable as well. Our proposed strategy for data harmonization consists of three steps. First, a unitary construct is defined based on the existing literature, preferably on the theoretical framework surrounding the construct. Second, the various instruments used to measure the construct are evaluated as operationalizations of this construct, and retained or excluded based on this evaluation. Third, the scores of the included measures are recoded on the same metric. We illustrate the use of this method with three example constructs focal to the Collaboration on Attachment Transmission Synthesis (CATS) study: parental sensitivity, child temperament, and social support. This process description may aid researchers in their data pooling studies, filling a gap in the literature on the first step of data harmonization.

• Data harmonization in studies using combined datasets is of vital importance for the validity of the study results.
• We have developed and illustrated a strategy on how to define a unitary construct and evaluate whether instruments are operationalizations of this construct as the initial step in the harmonization process.
• This strategy is a transferable and reproducible method to apply to the data harmonization process.

## Graphical abstract

**Figure:**
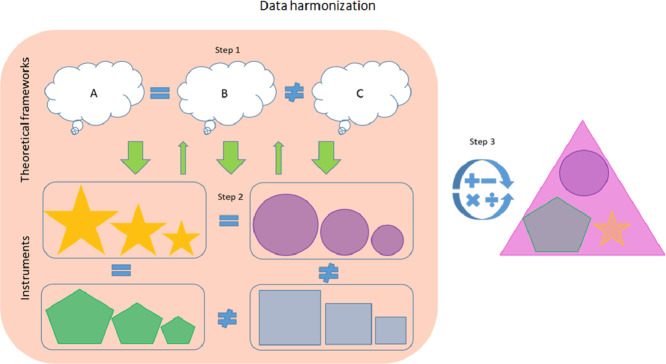

Specifications Table

**Table:** 

Subject Area:	Psychology
More specific subject area:	Developmental psychology
Method name:	Conceptual comparison to assure construct validity in data harmonization
Name and reference of original method:	Not applicable
Resource availability:	Not applicable

## Method details

### Background

In projects that pool individual participant data (IPD) for secondary data analysis, harmonization of the variables that were measured across the various studies is a vital step to ensuring that researchers are not combining different constructs as if they were similar in their analyses. Although there are many methods for harmonization currently described in the literature (e.g., [31,^32^,40]), these methods primarily focus on the final step of harmonization: the restructuring of measurements resulting from different instruments to measure a single construct in the same format. However, a necessary condition for harmonization is that those measures must operationalize the same underlying construct. This harmonization is done on different levels, as measures could operationalize very similar constructs (e.g., different varieties of apples are all caught under the construct of ‘apple’), but measures could also operationalize constructs that are similar on a higher level (e.g., apples and oranges are both fruits). The process of harmonization includes decisions on the level on which constructs are harmonized, and which measures cannot be included on this level (e.g., potatoes are food, but not fruits, which is our level of interest: therefore potatoes are excluded). It is crucial to be precise about this conceptual comparison of measurements, because the validity of study conclusions depends on the validity of the constructs that are measured [24]. We have developed a strategy for defining constructs and comparing measures in order to determine their common denominator as a first step in the harmonization process. This strategy combines a top-down approach to evaluating the theoretical constructs underlying the measures and a bottom-up approach to evaluating measures prior to the recoding of values or categories. In this paper, we outline this strategy and illustrate our harmonization of three constructs (parental sensitivity, child temperament, and social support) as part of the Collaboration on Attachment Transmission Synthesis (CATS) project [64]. The CATS-project is an IPD meta-analytic investigation focused on synthesizing the literature on the association between parental state of mind regarding attachment and the child-parent attachment relationship. The CATS database holds pooled data from a large number of studies on this association, as well as their data on demographics, psychological characteristics, and family functioning that were collected in the original studies. The aim of the project is to examine the mechanisms behind, and the boundaries of, the association between adult attachment state of mind and child-parent attachment relationship quality by leveraging moderating and mediating variables. The variables of parental sensitivity, child temperament, and social support figure centrally in the conceptual model guiding CATS as potential mediators and moderators.

### A 3-Step Strategy of IPD Data Harmonization

1. *Defining a unitary construct*. This step represents the top-down strategy in our approach. In order to harmonize each construct, we began by familiarizing ourselves with the theoretical framework surrounding the construct by conducting a review of the existing literature. Our priority was on handbooks, theoretical articles, position papers, round table discussions, and literature reviews, although in absence of these, empirical papers were used as well. We mapped the theoretical framework and evaluated whether one dominant framework or multiple frameworks existed. If there was no consensus in the literature, we assessed whether there was overlap for parts of the theory in the different frameworks by sifting through the theoretical dimensions and comparing these across frameworks. If there appeared to be overlap, we examined whether the dimensions in the different frameworks might be different names for the same underlying construct. We specified the overlapping dimensions at the lowest possible order of abstraction to avoid losing information, but used higher-order data if overlap could only be seen on a higher level (i.e., when subdimensions differed between theoretical frameworks, but higher dimensions were similar, e.g., that apples and oranges are both fruits).
  It was sometimes necessary to go back and forth between this step and the second step (described below) of evaluating the instruments used to measure the construct, because the availability of the data also impacted decision-making in the first step. For instance, when all instruments providing data for our study were developed based on the same theoretical framework, it was not necessary to assess the overlap with other theoretical frameworks.
2. *Evaluating the instruments used to measure the construct*. This step represents the bottom-up strategy in our approach. For each of the instruments that were used to measure the construct in our dataset, we determined its theoretical basis and which parts of the theory it measured. If only part of the instrument was in line with the framework, only this part was retained. We were again as detailed as possible by assessing which theoretical subdimensions were measured by which subscale or even item(s). However, this process was a balancing act between precision and availability. For example, we gave priority to including a less specific subscale if it led to a substantial increase in the available data, for instance when another often-used instrument could only be harmonized on a higher level of abstraction. This way, we tried to include as much data as possible with as high a convergent validity as possible.
  Sometimes, a construct is not related to any distinct theoretical framework. In such a case, we focused our conceptual comparison on the instruments to determine whether they measured the same construct. This was done by reading instrument manuals, validation articles, methods sections of empirical articles using the instruments, and review articles comparing the instruments.
3. *Recoding the scores*. Once it was decided which (sub)scales or items could be retained based on steps 1 and 2, the final step of the harmonization process was choosing a method for the recoding of scores to the same metric based on the existing literature [31,^32^,40]. Previous harmonization efforts often used *z*-scores, *t*-scores or category-centered scores for standardizing scores of different instruments [32], but this approach was not feasible, due to the lack of population norm scores for the instruments used to measure the constructs of interest. Standardization based on sample distributions would conflate mean level differences between study samples and individual variance (i.e., a score of 3 on caregiver sensitivity would get a positive *z*-score in a sample with on average low levels of caregiver sensitivity and a negative *z*-score in a sample with on average high levels of sensitivity). We therefore chose the simple calibration method [40], which transforms continuous measures to operate on the same scale through a calibration model. Specific calibration models are described per construct below. We compared scale score descriptions between instruments and matched them between instruments. Likert scales were recalculated to the same metric as the ‘gold standard’ measure or, if there was no gold standard measure, to the largest range used by any instrument measuring this construct.

### Illustrating the data harmonizing strategy

Our three-step strategy for defining the unitary constructs and harmonizing the measures used to operationalize them for three central constructs in the CATS IPD database (parental sensitivity, child temperament, and social support) is illustrated below. Unique data harmonization challenges for all three constructs were encountered. As such, we also describe our mitigation approaches to overcome these challenges.

#### Parental sensitivity

*Step 1*. We began by searching for literature reviews and meta-analyses on parental sensitivity, with a focus on papers that solely emphasized this construct and related constructs. Several meta-analyses [21,66] and literature reviews [43] were found. These all indicated that the term ‘parental sensitivity’ derived from the theoretical framework of attachment theory. It was defined by Ainsworth, Bell, and Stayton [1] as “a parent's ability to (1) notice child signals, (2) interpret these signals correctly, and (3) respond to these signals promptly and appropriately”. The scale to score maternal sensitivity/insensitivity was part of the broader Maternal Care scales. It was designed to assess individual differences in parental (originally maternal) behaviors as a predictor of the quality of the mother-child attachment relationship. A key aspect of the scale is, in line with the definition, appropriate responding, where appropriate meant that the distress of the child was effectively alleviated by the response of the parent. The construct of sensitivity was therefore meant as a reciprocal process between the child and the parent, with the child signaling, the parent noticing, interpreting, and promptly responding, and then the child having his or her needs met. The framework of attachment theory is the dominant framework for this concept. There are no further subdimensions, with sensitivity itself being a subscale of the Maternal Care scales [2].

*Step 2*. Sensitivity was measured with eight different instruments across the individual studies included in the CATS IPD database: the original Ainsworth Sensitivity scale, Emotional Availability Scale (EAS 3rd and 4th edition; [9,10]), Maternal Behavior Q-Sort (MBQS; [46]), CARE-Index [17], Parent Child Observation Guide (PCOG; [8]), NICHD Study of Early Child Care and Youth Development (SECCYD) sensitivity scales [45], Infant Caregiving Scales [33], and Coding Interactive Behavior (CIB; [23]). The systematic review of focal measures of “sensitivity” by Mesman and Emmen [43] was instrumental in this step in terms of evaluating the fit between the instruments that were used and the construct of parental sensitivity. Specifically, the review by Mesman and Emmen compared commonly used observational instruments to assess parental sensitivity to the original Ainsworth sensitivity scale. Five of the alternative instruments were included in the review (EAS [9], [10], MBQS [46], CARE-Index [17], NICHD SECCYD sensitivity scales [45], and CIB [23]) and these were all developed with Ainsworth's original sensitivity construct in mind. Most of the instruments have a sensitivity subscale (as was the case for the original sensitivity scale as part of the Maternal Care scales) or a sensitivity composite score made up of several subscales. The MBQS [46] yields a single sensitivity score as the only outcome of the instrument. The review by Mesman and Emmen [43] described the NICHD SECCYD sensitivity scales [45] and the MBQS [46] as the instruments measuring sensitivity most similarly to the original construct definition. The other sensitivity scales (EAS [9], [10], CARE-Index [17], and CIB [23]) were slightly broader than the original Ainsworth construct, as these included items on positive affect and warmth displayed by parents. Mesman and Emmen [43] concluded in their review that the instruments all include the main elements of the original sensitivity scale devised by Ainsworth et al. [1]. However, it has also been noted that it is better to separately assess sensitivity and positive affect/warmth, because while expressing warmth is different among cultures, sensitivity as adequately responding is universal [1]. Studies also show mixed results on the association between positive affect/warmth and parent-child attachment. Excluding the items of the EAS [9], [10], Care-Index [17], and CIB [23] referring to positive affect/warmth was not possible in our dataset, because either these observational scales provided overall global scores for sensitivity based on scale descriptions or because we did not have data on the item-level. We included the data from these instruments in the CATS dataset, because this makes it possible to perform sensitivity analyses with and without these data to assess if results are similar between the strict definition of sensitivity and the operationalization of sensitivity including positive affect and warmth. Performing sensitivity analyses keeps researcher degrees of freedom constrained. Furthermore, exclusion of these data would have led to the loss of over 500 parent-child dyads, which makes the dataset less generalizable and statistically powerful.

We next evaluated the two instruments that were not included in the review by Mesman and Emmen [43]: the Infant Caregiving Scales [36] and the Parent Child Observation Guide [9]. The Infant Caregiving Scales were developed based on the descriptions of the Ainsworth Maternal Care scales and a sensitivity subscale was derived from this measure with the help of expert judges. The items in the sensitivity subscale do not hold any reference to positive affect or warmth. Given the overlap with the original sensitivity scale by Ainsworth et al. [1], we included this instrument in our harmonized sensitivity construct. The Parent Child Observation Guide was developed as a parent-child interaction assessment instrument, which was not clearly grounded in the theoretical framework of attachment and sensitivity as defined by Ainsworth et al. [1]. The instrument is used to assess both the parent's and the child's behavior by coding whether certain behaviors did or did not occur during the observation period. For parents, the items loaded on a sensitivity subscale or a teaching subscale that were both found to predict child cognitive and social competence [8]. Given the lack of grounding in attachment theory, the event coding of behaviors without taking into consideration the appropriateness of the responses that is vital to the Ainsworth sensitivity coding, and the lack of attachment as a reported outcome, we excluded this instrument from our sensitivity construct.

Several studies have measured parental sensitivity on multiple occasions. If this was the case, we used the data from the timepoint that was in between the measurements of our independent and dependent variable, so that parental sensitivity could be tested as a mediator variable. If multiple measurements were done within this timeframe, we prioritized conceptual similarity of the measurements to the Ainsworth sensitivity scale. If the same instrument was used at multiple eligible timepoints, we chose the timepoint at which the data was most complete. If the data were equally complete at multiple timepoints, we chose the timepoint that was closest to the ‘middle’ between the measurements of the independent and dependent variable.

*Step 3*. Ainsworth et al. [2] were the first to conceptualize sensitive responsiveness and operationalize this construct as an observer rating scale. As included in the Maternal Care scales, the rating scale for sensitivity ranged from 1 to 9. This was used as a reference scale for the recoding of the scores measured with other instruments. We recalculated scores on the other instruments with Likert-scales to 9-point scales using the arithmetic equivalents in Table 1.

**Table 1:** Conversion formulas for parental sensitivity data.

Likert-scale	Which instruments?	Conversion formula
1-9	Ainsworth scale [2], EAS 3rd edition [9]	none
1-4	NICHD SECCYD sensitivity scale, 15-month system [45]	(score - 1) * 2.67 + 1
1-5	CIB [23]; NICHD SECCYD sensitivity scale, 24-month system [45]	(score - 1) * 2 + 1
1-7	EAS 4th edition [10]; Infant Caregiving Scales [33]	(score - 1) * 1.33 +1
0-14	CARE Index [17]	score * 0.571 + 1

**Fig. 1:**
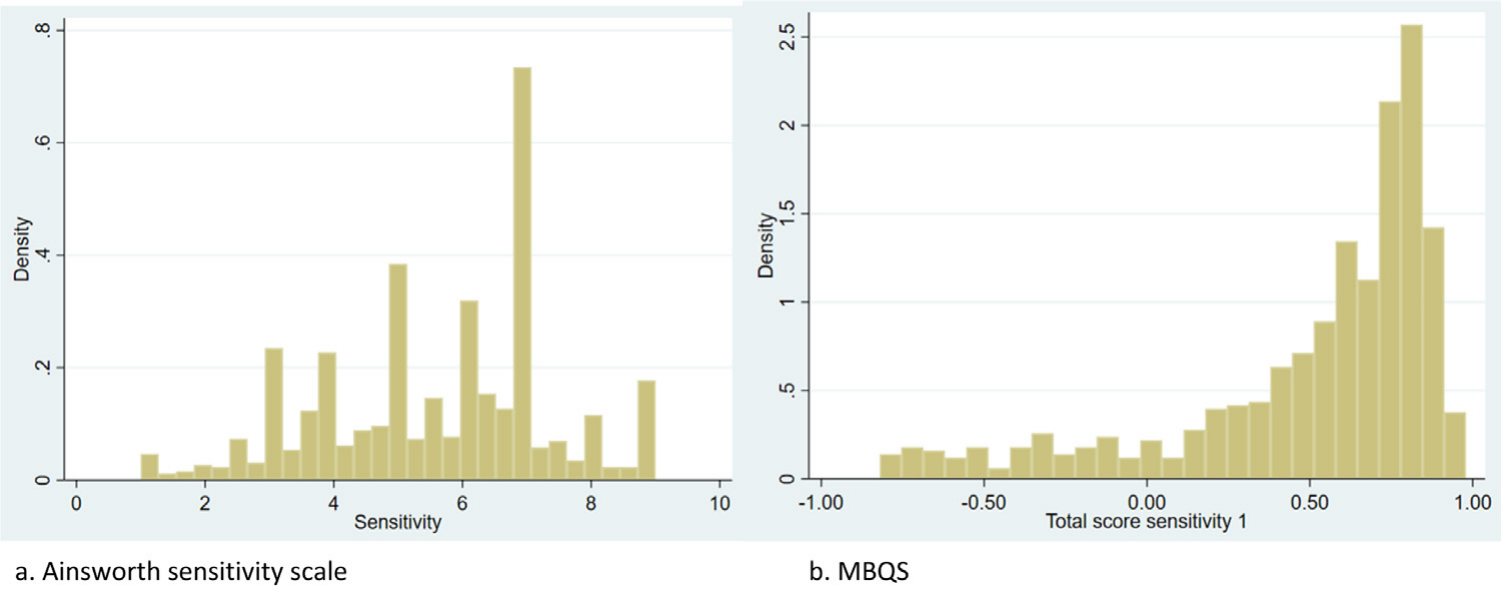
Distributions of parental sensitivity scores measured by Ainsworth scale (1a) and MBQS (1b).

The MBQS was measured on a continuum from -1 to 1 and therefore could not be recalculated in this way. If we could assume that the same underlying construct of sensitivity was measured both with the Ainsworth sensitivity scale and with the MBQS, as we argued in Step 2 of the harmonization strategy, then the distribution of scores should also be similar between measures, at least if the groups in which they were used were comparable. We therefore set out to compare the populations in which the Ainsworth sensitivity scale and the MBQS were used, and the resulting score distributions. Results showed that the population in which the Ainsworth sensitivity scale was used was less often considered ‘at-risk’ (38%, *N* = 943) than the population in which the MBQS was used (51%, *N* = 759). Populations were thus not entirely comparable, because one would expect lower sensitivity scores in at-risk populations. However, the histograms of each measure (Fig. 1a and b) showed that the distribution of the MBQS was highly negatively skewed, whereas the scores on the Ainsworth sensitivity scale were relatively normally distributed. For all other instruments, distributions were (near) normal as well, making it plausible that ‘sensitivity’ as a construct is normally distributed, while the Q-sort method used in the MBQS left more room for the low end of the scale, thus leading to a large tail in the distribution. We therefore ‘forced’ the distribution of the MBQS scores to look similar to the distribution of the Ainsworth sensitivity scores by transforming the scores.

**Fig. 2:**
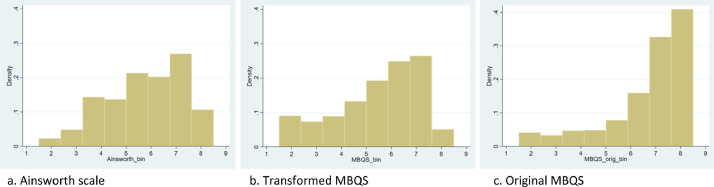
Distributions of binned parental sensitivity scores.

Several transformation methods were employed and negative reciprocal transformation of the scores (-1/score) proved most successful. Given that the scores of the Ainsworth sensitivity scale were on a scale from 1-9, we then proceeded with ‘binning’ the transformed MBQS scores (ranging from 0.4 to 1) in 8 equally wide bins ranging from 1.5 to 8.5. We did the same with the original MBQS scores and the Ainsworth scores, to facilitate comparison. Fig. 2 shows the distributions of the binned scores. The transformed MBQS scores were a better fit to the Ainsworth distribution of sensitivity scores than the untransformed scores. The distributions showed that the mean (5.85 and 5.53, respectively), standard deviation (1.78 and 1.96, respectively) and the quartiles (4.5, 6.5, 7.5 and 4.5, 5.5, 7.5, respectively) of the Ainsworth sensitivity scores and the transformed MBQS scores were roughly the same, whereas this was not the case with the untransformed MBQS scores (*M* = 6.93, *SD* = 1.87, quartiles are 6.5, 7.5, 8.5). This similarity justified harmonizing the MBQS using the bins derived from the reciprocal transformation.

As a final check, we performed sensitivity analyses to compare scores retrieved from strict and broader operationalizations (i.e., including positive affect and warmth) of the parental sensitivity construct. Specifically, scores derived from the Ainsworth scales, NICHD SECCYD sensitivity scale, MBQS, and Infant Caregiving Scales were compared to scores derived from the EAS, Care-Index and CIB. Given that we had no overlap in scores based on different instruments in each study, we could not use correlations between scores to assess their similarity. Furthermore, given that study samples were very diverse (i.e., ranging from normative samples to samples including parents with severe mental health problems, and sampled from 10 different countries), descriptive statistics of scores derived from the strict and broader operationalizations of parental sensitivity could not be directly compared either. We therefore examined the associations between two operationalizations of parental sensitivity and the main outcome measure in the CATS study: child-parent attachment security (operationalized as secure/insecure). To compare odds ratios across categories of strict/broader operationalizations, we tested for interactions between parental sensitivity scores and strict/broad operationalization in a multilevel logistic regression model including attachment security as a dependent variable and study as grouping variable. The results showed no significant interaction effect (OR = 0.99, *p* = .926), meaning that there was no empirical reason to differentiate between the strict definition of sensitivity (OR = 1.24, 95% CI 1.14-1.35) from the operationalization of sensitivity including positive affect and warmth (OR = 1.24, 95% CI 1.08-1.44). These results justified the harmonization of both operationalizations of parental sensitivity as representing the same construct.

#### Child temperament

*Step 1*. Our search started with the Handbook of Temperament [67], a key source of information about temperament. The chapter on models of child temperament [42] distinguishes five theoretical models of child temperament:

(1) the behavioral styles approach of Thomas and Chess, that focused on ‘the stylistic component of behavior, that is, the *how* of behavior’ ([27], p. 508). They identified nine categories of behavior (activity level, regularity, adaptability, approach-withdrawal, threshold of responsiveness, intensity of reaction, quality of mood, distractibility, attention span) and three types of children based on their temperamental characteristics (easy, difficult, and slow-to-warm-up).
(2) the criterial approach of Buss and Plomin, that defines temperament as “a set of inherited personality traits that appear early in life” ([27], p.508) This initially led to 4 temperamental dimensions: Emotionality, Activity, Sociability, and Impulsivity.
(3) the psychobiological approach of Rothbart, that sees temperament as “constitutionally based individual differences in reactivity and self-regulation, in the domains of affect, activity and attention” ([53], p.100). This approach has identified three broad dimensions of temperament: negative affect, surgency, and effortful control.
(4) the emotion regulation model of Goldsmith and Campos, which defines temperament as “differences in the experience and expression of emotion, including their regulatory aspects” ([28], p. 2). It focuses on individual differences in the primary emotions, such as joy, interest, sadness, anger, and fear.
(5) the behavioral inhibition model of Kagan [35], that focuses solely on behavioral inhibition, not on the other dimensions of temperament. Importantly, none of the data within our dataset were measured with instruments based on this approach.

In a seminal roundtable with all originators of the theories except Kagan, the theorists agreed that activity level and emotionality were part of temperament, but consensus could not be reached for any other of the dimensions [27]. Each theoretical approach is accompanied by their own instruments to assess temperament. During the decades after this roundtable, factor analytic work was done with these instruments to establish the structure of temperament, both in infancy [26,30] and childhood (e.g., [29,42]). In 2006, Rothbart, original theorist of the psychobiological approach, and Bates, originator of the ICQ instrument based on the Thomas and Chess approach, co-authored a chapter on temperament. They drew two conclusions regarding the structure of temperament from the factor analytic studies: (1) that the “structure of temperament corresponds more to dimensions of reactivity than to a general style” (p. 104), meaning that temperament can differ across dimensions within a person, and (2) that there seem to be three (possibly four) broad factors of child temperament: Surgency/Extraversion, Negative Emotionality, Effortful control/Task persistence, and possibly Agreeableness/Adaptability.

With these four broad factors of temperament determined, we turned to our research questions to determine whether our focus should be on any temperament factors in particular. The CATS project aimed to examine the role of child temperament as a differential susceptibility factor (i.e., factor that makes some children more susceptible to their environment than others) of the association between parental sensitivity and parent-child attachment. Previous studies using temperament as differential susceptibility factor have focused mainly on the general factor of negative emotionality or more specifically on inhibition (fear) and irritability (anger), which are smaller aspects of this broad negative emotionality factor [62]. The meta-analysis by Slagt et al. [57] also showed that children higher on negative emotionality were both more vulnerable to negative parenting as well as profiting from positive parenting during infancy. We therefore focused on this broad factor with the two subcomponents (i.e., fear and irritable distress). Focusing on two distinct subcomponents and one broad factor that combines the two allows for sensitivity analyses comparing the effects of these temperament operationalizations. For the purpose of the data harmonization, we define Negative emotionality as “a general tendency to experience negative emotions” [42].

*Step 2*. Temperament was measured with 9 different instruments across the individual studies included in the CATS project: Infant Behavior Questionnaire (IBQ; [51]), IBQ-Revised (IBQ-R; [26]), IBQ Very Short Form (IBQ-VSF; [49]), Infant Characteristics Questionnaire (ICQ; [6]), Behavioral Style Questionnaire (BSQ; [41], Early Childhood Behavior Questionnaire (ECBQ; [48]), Childhood Behavior Questionnaire (CBQ; [54]), Short Temperament Scale for Toddlers (STST; [47]), Questionnari Italiani del Temperamento (QUIT; [4]), Emotionality Activity Sociability Temperament Survey for Children (EAS; [12]). These instruments were derived from three different theoretical frameworks, see Table 2 below.

**Table 2:** Comparison of temperament instruments in the CATS dataset.

Instrument	k studies / N participants	Theoretical framework	Scale ‘Negative emotionality’	Subscale ‘Fear’	Subscale ‘Irritable distress’
IBQ	7 / 738	Rothbart	Negative Affectivity composite	Fear / distress to novelty	Distress to limitations
IBQ-R	3 / 108	Rothbart	Average score of Fear and Distress to limitations scale	Fear	Distress to limitations
IBQ-VSF	1 / 200	Rothbart	Negative emotionality	-	-
ICQ	8 / 565	Thomas & Chess	Average score of Unadaptable and Fussy-Difficult	Unadaptable	Fussy-Difficult
BSQ	1 / 40	Thomas & Chess	Average score of Approach and Mood	Approach	Mood
ECBQ	1 / 67	Rothbart	Average score of Fear/Shyness and Frustration	Average score of Fear and Shyness	Frustration
CBQ	1 / 24	Rothbart	Average score of Fear and Anger/Frustration	Fear	Anger/Frustration
STST	1 / 104	Thomas & Chess	Average score of Approach and Reactivity/Cooperation	Approach	Average score of Reactivity and Cooperation
QUIT	1 / 40	Thomas & Chess	Average score of Novelty inhibition and Negative emotionality	Novelty inhibition	Negative emotionality
EAS	1 / 148	Buss & Plomin	Average score of Shyness and Emotionality	Shyness	Emotionality

The IBQ variants [28,52,54] and the ICQ [6] are most commonly used in the CATS dataset (10 and 9 times, respectively), with all other instruments only being used in one study. We therefore first set out to compare the subscales involved in the broad factor negative emotionality and the components of fear and irritable distress within the IBQ variants and the ICQ (see also Table 2). The IBQ [54] stems from the Rothbart theoretical framework and assesses 6 subdimensions of infant temperament: activity level, soothability, fear (sometimes called ‘distress to novelty’), distress to limitations, smiling and laughter, and duration of orienting. The subdimensions of ‘Fear’ and ‘Distress to limitations’ (i.e., irritable distress) together constitute a composite measure of “Negative affectivity” [52], which reflects Negative emotionality as defined above.

The revised version of the IBQ, the IBQ-R [26], was expanded to include 14 subscales: approach, vocal reactivity, high pleasure, smile/laughter, activity level, perceptual sensitivity, sadness, distress to limitations, fear, falling reactivity/rate of recovery from distress, low pleasure, cuddliness, duration of orienting, and soothability. The composite measure of “Negative affectivity” includes four subscales (Sadness, Distress to Limitations, Fear, and Falling Reactivity/Rate of Recovery from Distress) and was thus too broad for our definition of negative emotionality. We therefore combined the subscales ‘Distress to limitations’ and ‘Fear’ to reflect negative emotionality as we defined it. The same subscales were used separately as measures of irritable distress and fear.

The IBQ-VSF [52] has three subscales: negative emotionality, positive affectivity/surgency, and orienting/regulatory capacity. The “Negative emotionality” subscale reflects the broad dimension we aim for (although slightly broader as it also includes items included in the ‘Sadness’-scale of the IBQ-R), but this short questionnaire does not provide separate scale scores for fear or irritable distress. Therefore, we used this slightly broader subscale in our analyses.

The ICQ [6], in line with the Thomas and Chess theoretical framework, assesses infant difficultness. It has 4 subscales, namely ‘Fussy-difficult’, ‘Unadaptable’, ‘Dull’, and ‘Unpredictable’. The ‘Fussy-difficult’ subscale measures the general mood, intensity and frequency of fussing and crying, how easily children are upset, and soothability. This subscale reflects the ‘irritable distress’ component of negative emotionality. The ‘Unadaptable’ subscale measures reactions to novelty and disruptions and adaptation in general. This reflects the ‘fear’ component of negative emotionality.

Comparing the content of the IBQ and ICQ subscales, the ICQ scales seem slightly broader. The ICQ Fussy-Difficult scale focuses on more general distress and includes soothability, whereas the IBQ Distress to Limitations scale limits itself to distress in episodes where the child is confronted with restrictions. The ICQ Unadaptable scale focuses on more general adaptation, whereas the IBQ Fear scale is mostly aimed at fear in novel situations.

Given that we do not have ICQ item-level data in the CATS dataset, we went ahead with these subscales as reflecting the same underlying constructs of fear and irritable distress, but we will perform sensitivity analyses with the IBQ data and ICQ data separately to assess whether results are similar with the narrow operationalization of the IBQ and the slightly broader operationalization of the ICQ.

One study used both IBQ and ICQ to measure child temperament at the same timepoint. In this case, we used the IBQ data, because this is slightly more in line with the dimension of Negative emotionality and the subdimensions as mentioned in the theoretical framework.

Six studies in the CATS dataset used other instruments than the IBQ and the ICQ, though mostly from the same theoretical frameworks. Discussion to develop consensus about which instruments could and could not be harmonized took place between MLV and AH, after which these decisions were discussed again with CS. The ECBQ [51] is a Rothbart measure with similar structure as the IBQ, but for use with toddlers. It contains 18 subscales. The Frustration subscale is similar in content to the Distress to limitations subscale in the IBQ. The Fear subscale seems to be more limited, as it only includes inhibition/unease in situations of novelty and threat, but not in social situations. These situations seem to be captured in the Shyness subscale. Because these situations are included in the IBQ and the ICQ, we chose to average the scores on the Fear and Shyness subscales to include as our Fear component. The Negative Affectivity composite scale contains 8 subscales and is thus broader than the construct of our interest, we therefore combined the two components resembling Fear and Irritable distress to reflect our negative emotionality construct.

The CBQ [58] is also a Rothbart measure with similar structure, designed for use with children age 3 to 7 years. It has a Fear subscale and an Anger/Frustration subscale that assesses the same construct as the Distress to limitations subscale in the IBQ [54]. The Shyness subscale is also present in this measure, but, contrary to the factor analytic findings with the ECBQ, this subscale loaded on the Surgency dimensions and not on the Negative Affectivity dimension [54]. We therefore chose to use the Fear subscale without the Shyness subscale to best reflect our Fear component. The proposed Negative Affectivity composite encompassed more subscales than the two of our interest, therefore we combined the subscales ‘Anger/Frustration’ and ‘Fear’ to reflect negative emotionality as we defined it.

The STST [50] is an Australian adaptation of the Temperament Scale for Toddlers [25], which derives from the Thomas and Chess theoretical framework. It has six subscales: approach, reactivity, rhythmicity, cooperation, persistence, and distractibility. The ‘Approach’-subscale measures the child's response to unfamiliar people and can be seen as similar to the Fear construct. The ‘Reactivity’-subscale measures fussiness and frustration level when restricted, but also more general activity level. The ‘Cooperation’-subscale measures how upset the child gets during routine activities (e.g., waiting for food, brushing hair/teeth). Although these two subscales include two items on general activity level, and are thus slightly broader than the irritable distress component of temperament, we combined them for use in our study.

The BSQ [44] is another temperament scale for use with older children. The 9 dimensions theorized by Thomas and Chess form the basis for the subscales of the questionnaire. The subscale ‘Mood’ measures the tone of overall affect (positive or negative) and is thus slightly broader than the IBQ subscale of distress to limitations to resemble the Irritable distress component of temperament. The subscale ‘Approach’ refers to the child's response to the environment, similarly to the STST described above, and thus reflects the Fear component.

The QUIT [4] is also derived from the framework of Thomas and Chess and has six subscales. The ‘Negative emotionality’ subscale describes the child's tendency to express negative emotions, which is similar to the component of irritable distress. The subscale ‘Novelty inhibition’ describes the child's tendency to respond with inhibition to novel stimuli, which reflects the component of fear.

The EAS [14] is the only instrument derived from the theoretical framework of Buss and Plomin. It contains four subscales (Emotionality, Activity, Sociability, and Impulsivity), one for each temperament dimension. The Emotionality subscale measures how easily the child gets upset or cries, which reflects the same underlying construct as the irritable distress component. The Shyness subscale measures social inhibition, which is similar to the Fear component, but limited to social situations (same as in the IBQ). However, we decided to use this slightly narrower subscale in our analyses.

One study used two instruments to measure temperament, the QUIT and the ITQ-R, which derive from the same theoretical framework. We ended up using the data collected with the QUIT, because the ITQ-R data was available for less than half of the sample.

As an assessment of the associations between subscales of these instruments, several studies have used multiple temperament instruments in one sample. One such study compared scales on the IBQ, ICQ, and RITQ ([30], as described in [53]). It found that distress to novelty (which we defined as ‘inhibition/fear’) was assessed by all these instruments (IBQ Fear, ICQ Unadaptable, RITQ Approach-Withdrawal scales). Intercorrelations were high: for mother average *r*=.64, for daycare teachers *r*=.63. Irritable distress was also measured by all instruments with the scales IBQ Distress to limitations, ICQ Fussy/Difficult, and RITQ Negative Mood (which includes positive mood at one pole) scales. Average intercorrelation for M was *r*=.54, for daycare teachers *r*=.71.

Another study by Lemery et al. [39] used principal components analyses with different questionnaires and showed the existence of four composite temperament dimensions, among which distress-anger and fear. For infants, IBQ Distress to limitations, ICQ Fussy, and ITQ Mood loaded on the distress-anger component. IBQ Distress to Novelty (fear), ICQ Adaptability, and ITQ Approach loaded on the fear component. For toddlers, the scales ICQ Difficult and BSQ Mood loaded on the distress-anger component, whereas ICQ Adaptability and BSQ Approach loaded on the fear component.

Mervielde & de Pauw [42] found clear correspondence between the scales EAS Emotionality and TTS Mood in early childhood (*r* = .60) and between BSQ Adaptability/Mood, EAS Emotionality, and CBQ Negative Affect in middle childhood (range *r* = .48 to *r* = .54). This is in line with how we structured the ‘Irritable distress’ component.

The results of these studies underline the overlap between the measures and support our choices in the harmonization process.

*Step 3*. The IBQ and ICQ as most often used instruments measured temperament on a 7-point scale. We chose to convert the scores on the other scales to a 7-point scale, also because this was the largest scale in use (other instruments had 5- or 6-point Likert scales). Arithmetic equivalents can be found in Table 3.

**Table 3:** Conversion formulas for temperament data.

Likert-scale	Which instruments?	Conversion formula
1-7	ICQ [6], IBQ [54], IBQ-R [28], IBQ-VSF [52], ECBQ [51], CBQ [58]	none
1-6	BSQ [44], STST [50], QUIT [4]	(score - 1) * 1.2 + 1
1-5	EAS [14]	(score - 1) * 1.5 + 1

Data from two studies had to be excluded, because the available scores were not in line with the rating scales of the instruments. One study used the ICQ, but had only ICQ dichotomous difficult vs easy temperament categorizations. The other study used the BSQ, but scores ranged from 0-2 instead of 1-6, and an explanation for these discrepancies could not be resolved.

Finally, we performed sensitivity analyses for different operationalizations of negative emotionality. First, we examined the correlations between the broad factor of negative emotionality, and the smaller subcomponents of fear and irritable distress. The correlations between negative emotionality and fear and negative emotionality and irritable distress were high, both *r* = .83. However, the correlation between fear and irritable distress was *r* = .38, suggesting that these two subcomponents assess overlapping as well as unique parts of the negative emotionality construct in the CATS dataset. This indicates that results may differ depending on the choice of the subcomponent. Therefore in subsequent analyses we always performed analyses with the broad factor of negative emotionality as well as with the two subcomponents fear and irritable distress separately.

As a second series of sensitivity analyses, we compared scores retrieved from the IBQ and the ICQ on the subcomponents fear and irritable distress, because the operationalization of both scales was slightly broader in the ICQ than in the IBQ (see above). To compare odds ratios across measures, we tested in two separate analyses the interaction between fear and distress and IBQ/ICQ in a multilevel logistic regression model including attachment security as a dependent variable and study as grouping variable. For fear, the results showed no significant interaction effect (OR = 1.24, *p* = .174), meaning that there was no empirical reason to differentiate between the IBQ (OR = 0.91, 95% CI 0.75, 1.11) and the ICQ (OR = 1.08, 95% CI 0.87, 1.34). For distress, results did not show a significant interaction effect either (OR = 0.95, *p* = .733), with ORs not differing between IBQ (OR = 1.03, 95% CI 0.84, 1.26) and ICQ (OR = 0.96, 95% CI 0.77, 1.19). Given these results, it is justified to include fear and distress derived from IBQ and ICQ as operationalizations of the same subcomponents.

#### Social support

*Step 1*. In searching for a theoretical framework for the concept of social support, we found several overview papers that described that social support is mostly used in research as a buffering factor to health outcomes during stressful events [22,^60^,65]. In most of the empirical work that examines effects of social support, it is not well-integrated with the research and theory on close relationships, but measured with simple measures, such as ‘general satisfaction with support’ and ‘number of close contacts’. As such, the concept of social support as used in research in the field of developmental psychology is not embedded in one theoretical framework; instead, researchers draw on different theoretical frameworks for the embedding of social support. For instance, some researchers link the importance of social support to attachment theory by stating that individuals seek proximity to close others in times of distress [22], whereas other researchers link it to coping theory, social learning theory, or attribution theory [65].

Not only does social support lack a unified theoretical framework, it also lacks agreement in its definition. A concept analysis identified 30 distinct definitions of the concept [65]. Social support may be better viewed as a meta-construct that can be subdivided into distinct conceptual components, such as support network resources, supportive behavior, and subjective appraisal of support [63]. Support network resources are defined as the contacts a person regards as supporting them. Supportive behavior refers to the specific behaviors that can be offered as support. Subjective appraisal of support is the subjective evaluation that a person makes about the support they receive from their support network. Most studies focus on narrow operationalizations of social support, probably due to the complex nature of the concept. This often causes a focus on one of the three conceptual components of support. Several studies have compared the valence of actual received support and the subjective appraisal of support (also termed perceived support or satisfaction with support) and found that perceived support was a better predictor of health outcomes than actual received support (e.g., [22,^36^,^50^,59]. Perceived support also seems to be more commonly measured in studies, as reported by Feeney and Collins [22], but also evident in the CATS dataset. We therefore focused on satisfaction with support in the CATS study.

In our search for theoretical framing and defining a unitary construct of social support, we learned that two more distinctions are to be considered: sources of support and type of support. Regarding the sources of support, we set out to review the literature on whether it mattered who provided the support in the context of parenting. Distinctions can be made between partner, family, friends, acquaintances, professionals, and strangers, but comparisons are rarely made. It is suggested that support from close others is more influential than support from less familiar others [22]. A meta-analysis examined the association between support by family (spouse or mother), support by a broader network (friends and community) and maternal interactions of adolescent mothers with their infants [14]. Analyses revealed similar medium-to-large effect sizes for support by family and support by a broader network. A study by Coan et al. [15] showed that adults who were subjected to a stressor showed less brain activation in threat-related areas when holding their partner's hand, and to a lesser extent when holding hands with a stranger, compared to when alone. These findings are in line with the premise of the Social Baseline Theory that the human brain assumes close proximity to a network of familiar others and that this proximity to others diminishes stress responses in the brain [7]. We therefore focused on support given by familiar persons, without differentiating any further.

Finally, we sought literature on types of support given in the context of parenting. The research distinguishing between types of support is scarce, but several types of support are mentioned (with slightly varying terms): emotional support, informational support, material support, and appraisal support [3,^11^,37]. All types of support had significant negative correlations with post-partum depression in first-time mothers [37], and emotional support, informational support, and material support were positively associated with parenting competence and parenting satisfaction in African-American teenage mothers [11]. A meta-analysis on the association between social support types and maternal behaviors and attitudes showed similar (moderate) effect sizes for each type of support [3]. All types of support appear to be equally important. Our harmonization thus focused on satisfaction with support by familiar persons, not making distinctions between types of support provided.

*Step 2*. Social support was measured with 14 different instruments across the individual studies included in the CATS project: Who Does What questionnaire (WDW; [16]), Social Support Questionnaire (SSQ; [56]), short form of the SSQ (SSQ6; [55]), Social Support Questionnaire (SSQ; [38]), Multidimensional Scale for Perceived Social Support (MSPSS; [68]), Social Provisions Scale (SPS; [20]), Dyadic Adjustment Scale (DAS; [58]), Secure Base Scoring System (SBSS; [19]), Family and Friends questionnaire [34], support and stress questionnaire [61], two satisfaction items [5], emotional support scale [13], observational assessment of co-parenting and spousal support [44] social support interview [18].

Instruments were all used in one or two studies. Given that there was not one or two instruments that were more common than other instruments, we compared each instrument to the criterion of measuring satisfaction with support by familiar persons.

Six instruments were explicitly focused on measuring satisfaction with support. The WDW [18] asks participants questions about the task divisions in three domains of family life, namely household tasks, family decisions, and childcare responsibilities. For each domain, questions are asked about how the task division is, how the ideal task division would be and how satisfied the participants were with the task division. The satisfaction items together formed a satisfaction scale, measured on a 5-point scale from very dissatisfied to very satisfied. The original SSQ [56] contains 27 items asking who a participant can count on for support. After each item, participants rate on a 6-point scale how very satisfied to very dissatisfied they are with the total support provided. The satisfaction items are averaged to obtain a total satisfaction score. The 6-item short form of the SSQ, the SSQ6 [59], has a similar structure, but with only 6 items. The SSQ developed by Leerkes and Crockenberg [38] has only 4 items and was designed to assess satisfaction with support by partners and others on several domains related to parenting. Satisfaction was measured on a 5-point scale ranging from very dissatisfied to very satisfied. We averaged the score to obtain a total satisfaction score. The support and stress questionnaire [66] consists of 15 items that present potential sources of support. Participants were asked whether they receive support from these sources. If so, participants filled out their satisfaction with the support from this source on a 5-point scale ranging from not satisfied to very satisfied. Satisfaction scores were averaged to obtain a total satisfaction score. The two satisfaction items used in the Bailey et al. study [5] measured satisfaction with support from the baby's father and current partner and satisfaction with support from parents and others. The 7-point scale ranged from not satisfied to very satisfied, and was averaged to obtain a total satisfaction score.

Two instruments were not developed to operationalize satisfaction with support, but a rational interpretation identifies the items as belonging to the universe of satisfaction with support. The MSPSS [74] consists of 12 items measuring the perceptions of social support by family, friends, and significant others. The items do not ask directly for the satisfaction with the provided support, but they aim to assess the perceived quality of support, e.g. “I get the emotional help and support I need from my family”. We therefore included the measure in our harmonization. The 7-point scale ranged from very strongly disagree to very strongly agree, and was averaged to obtain a total satisfaction score. The emotional support scale [15] was used for coding the quality and the extent of social support available to mothers based on interview questions. Interview questions were about the satisfaction with support and the consistency and quality of help given to the mothers. The measure was thus not aimed solely at measuring satisfaction with support, but it was prominently included in the interview questions, thus we included the measure in our harmonization. The emotional support scale was double-coded by trained coders on a 7-point scale, ranging from almost non-existent to excellent.

The other six instruments were excluded because these did not measure satisfaction with support. Two instruments, the SPS ([22]; 24 items on 6 subscales) and the Family and Friends questionnaire ([37]; 15 items), were excluded because the formulation of the items (e.g., ‘There are people I can depend on to help me if I really need it’) was more indicative of whether there was a support network (i.e., quantity) than of how satisfied the participant was with the support provided. Two other instruments, the SBSS [21] and the observational assessment of co-parenting and spousal support [44] were observational measures of support rated by independent observers. As such, these measured whether support was given and not perceived satisfaction with support. Furthermore, the DAS [63] measured dyadic adjustment of the couple and was therefore more a measure of relationship quality than of more general social support. Finally, the social support interview [20] asks about sources of support within the family and outside of the family. This instrument is aimed at measuring the quantity of support and was therefore excluded from the harmonization.

*Step 3*. Before deciding what scale to recode the scores to, we discussed whether the descriptions of the scale values all covered the same continuum of dissatisfaction to satisfaction with support. These descriptions varied from very dissatisfied to very satisfied (or vice versa), from very strongly disagree to very strongly agree, from not satisfied to very satisfied, and from almost non-existent to excellent (quality of support). First, we discussed how to compare the scale of disagree/agree with the scale of dissatisfied to satisfied. The disagree/agree answers were answers to questions regarding satisfaction, thus meaning ‘agree with satisfied’, which can be seen as similar to answering ‘satisfied’ to a question on the level of satisfaction. In a similar vein, the scale ranging from almost non-existent to excellent answered questions about the perceived quality of support, which was therefore also comparable to the continuum from dissatisfied to satisfied. Furthermore, ‘not satisfied’ on the lower end of a ‘not satisfied to very satisfied’-scale cannot compare to the statement ‘not dissatisfied/not satisfied’ as the neutral midpoint of a scale spanning the continuum from very dissatisfied to very satisfied. We thought it more likely that someone answering ‘not satisfied’ on the first scale, would answer at least ‘dissatisfied’ on the latter scale. We therefore decided that these various descriptions could all be considered to be on the same continuum.

We recoded scale scores to the scale with the largest score span (7 points), given that there was not one predominantly used scale. Arithmetic equivalents can be found in Table 4.

**Table 4:** Conversion formulas for social support data.

Likert-scale	Which instruments?	Conversion formula
1-7	Emotional support scale [15], two satisfaction items [5], MSPSS [74]	none
1-6	SSQ [60], SSQ6 [59]	(score - 1) * 1.2 + 1
1-5	WDW [18], SSQ [41], support and stress questionnaire [66]	(score - 1) * 1.5 + 1

### Conclusion

This article illustrated the detailed strategies involved when harmonizing datasets in IPD syntheses. This initial step of defining a unitary construct and evaluating whether measurements derived from different instruments are operationalizations of this construct is crucial to ensure the validity of the measured constructs, and thus for the validity of study conclusions [24]. The paper provides researchers with an aid for conducting data pooling of studies, while also filling a gap in the literature on data harmonization. We recommend using the strategies provided herein, along with existing articles focused on the restructuring of measurements from different instruments into the same format (i.e., the final step in the data synthesis process) to effectively and accurately derive an IPD dataset for analysis.

## Declaration of Competing Interests

The authors declare that they have no known competing financial interests or personal relationships that could have appeared to influence the work reported in this paper.
